# Injury-induced fetal reprogramming imparts multipotency and reparative properties to pericardial adipose stem cells

**DOI:** 10.1186/s13287-018-0959-1

**Published:** 2018-08-13

**Authors:** Jianfeng Tang, Xiaoming Wang, Kezhe Tan, Hongtao Zhu, Youming Zhang, Weili Ouyang, Xueqing Liu, Zhaoping Ding

**Affiliations:** 1 Department of Cardiology, Danyang People’s Hospital, West Xinmin Rd. 5, 212300 Danyang, People’s Republic of China; 2 Department of Cardiothoracic Surgery, BenQ Medical Center, Hexi Rd. 17, 210019 Nanjing, People’s Republic of China; 3 Department of Anesthesiology, Changhai Hospital, Naval Medical University, Changhai Rd. 168, 200433 Shanghai, People’s Republic of China; 4 Institue of Molecular Cardiology, Heinrich-Heine University of Düsseldorf, Moorenstr. 5, 40225 Düsseldorf, Germany

**Keywords:** Wilms’ tumor factor 1 (WT1), Pericardial adipose stem cells (pADSC), Pericardial fluid, Hepatocyte growth factor (HGF), Apoptosis, Angiogenesis

## Abstract

**Background:**

Injury may induce a sequential activation of intrinsic reparative activity that supports the maintenance of tissue homeostasis.

**Method:**

In the present experiments, we investigated whether myocardial infarction (MI) was able to reinstate the expression of Wilms’ tumor factor 1 (WT1) as a key hallmark of fetal reprograming in the pericardial adipose-derived stem cells (pADSC). We characterized the immunophenotypical markers, cardiac potential, and reparative activity of WT1-expressing pADSC (WT1^pos^) isolated MI Wistar rats with an intact pericardial sac in which cardiac transudate was accumulated, sampled, and analyzed.

**Results:**

The WT1^pos^ cells formed colony-like aggregates in culture that subsequently generated phase-bright cells that homogenously constituted WT1 expression (> 98%). The WT1^pos^ cells shared identical surface markers with canonical pADSC, but enhanced transcripts for cardiogenesis (isl-1, gata-4, Sox2 and Tbx18) as well as cardiac commitment (endothelial: 28%; cardiomyogenic: 12.3%) in defined conditions. Remarkably, cardiac transplantation of WT1^pos^ cells promoted regional angiogenesis and myogenesis which led to significant functional amelioration of the infarcted hearts. Furthermore, we demonstrated that WT1^pos^ cells uniquely secreted hepatocyte growth factor (HGF) as a key antiapoptotic factor that promotes cardiac repair.

**Conclusion:**

Injury-associated fetal reprogramming in pADSC facilitates cardiac differentiation and promotes the reparative activity by enhancing HGF production. As such, injury-“conditioned” pADSC may represent a useful autologous cell donor from infarcted patients for cell-based therapy.

## Background

Heart failure caused by the death of cardiac muscle cells due to an insufficient blood supply to the heart remains as one of the major causes of mortality in Western countries. While a fish can generate new heart muscle cells and fully restore function after amputation [1], mammals are not so fortunate [2] with damage to the heart muscle resulting in tissue scarring and limited regeneration of muscle cells [3]. Investigators have long pursued strategies for replacing the lost heart muscle by transplanting stem cells either from bone marrow, blood, skeletal muscle, fat tissue, or from the heart itself into the damaged heart [4, 5]. Among the donor cells tested so far in preclinical and clinical studies, cardiac stem cells located on the heart surface, so-called epicardial-derived progenitor cells (EPDC), have emerged as an attractive source with significant cardiac potential arising from their embryonic origin [6]. Notably, an important feature of adult EPDC is their capability for redeployment of their developmental program in response to injury stimulation by re-expression of Wilms’ tumor factor 1 (WT1) as an important genetical feature for embryonic cardiogenesis [7, 8]. This unique characteristic has therefore brought about great clinical interest in using EPDC as donor cells to reconstitute the damaged heart after ischemic injury. Animal studies have already demonstrated that EPDC contributed to de novo formation of cardiomyocytes [9], and implantation of EPDC into the infarcted mouse heart efficiently preserved cardiac contractile function and prevented cardiac remodeling after injury [10].

Recently, the pericardial tissue was also recognized to consist of a pre-epicardial origin of mesenchymal stem cells, so-called cardiac colony-forming unit fibroblasts (cCFU-Fs), that give rise to all mesodermal lineages, including smooth muscle, bone, cartilage, adipose, endothelial, and heart muscle cells [11]. The epicardial stem cells can be isolated and expanded in vitro and favor a myogenic differentiation and, when transplanted into the infarcted heart in vivo, yield more effective cardiac repair in comparison with cells from subcutaneous fat [12]. However, it remains elusive whether the pericardial progenitors residing in the pericardial tissue could recapitulate an embryonic program in response to cardiac injury [8]. This question is of practical interest since pericardial adipose tissue is abundant and clinically easy to access, and thus the re-activated pericardial adipose-derived stem cells (pADSC) may represent one of the most suitable cell donors for cell-based therapy for cardiac ischemic disease [13].

Injury-induced repair is an intrinsic ability that supports the maintenance of tissue homeostasis, and includes a sequential activation and re-expression of specific transcription factors that shift the quiescent stem cells into a transient, activated state unique to regeneration [14]. It has been found that in multiple organs, such as the lung, gut, pancreas, mammary gland, skin, and liver, certain type of cells, defined by location, mitotic activity, and marker profile, are capable of multipotency [15]. In many cases, a population of reserved or relatively dormant stem cells that activate only in response to severe tissue damage exists alongside a pool of active stem cells [14]. Upon injury to the olfactory epithelium, for instance, the horizontal basal cells shift en masse to an activated, transient state characterized by the rapid initiation of the wound-response transcriptional program, which includes upregulation of differentiation-associated intermediate filament keratins and the stratified epithelium secreted peptides complex [16]. These genes are associated with epidermal differentiation and are involved in mitigating environmental stressors, promoting cell migration and shape changes, and establishing the barrier function of the epidermis [16]. In the epicardial cells, activation of the fetal reprograming process, characterized by the redeployment of WT1 expression, was dedicated to cardiac repair by cellular replacement [9] and paracrine support for neoangiogenesis [17] after myocardial infarction (MI).

Our attention was therefore drawn to the potential reactivation of fetal gene expression in the pericardial tissue. A working hypothesis of the present study is that the mitotically quiescent pADSC, under stressed conditions after cardiac injury, are subject to ongoing stimulation by the bioactive molecules present in the pericardial fluid [7] that has been shown to reprogram c-kit^+^ EPDC into an embryonic plasticity [18] and reinstate the transcription factor WT1 which serves as the master regulator of pADSC identity and reserved status. Moreover, we propose that WT1-expressing pADSC may exhibit more pronounced reparative activity than quiescent cells. Here, we show that injury-associated expression of fetal genes in pADSC potentiated cardiac repair through the proangiogenic and antiapoptotic activities of hepatocyte growth factor (HGF) released by WT1-positive pADSC (WT1^pos^).

## Methods

### Animal experiments

All the experiments were approved by the Institutional Animal Care and Use Committee at the Zhejiang Chinese Medical University (SCXK Hu 2008–0016) and conducted in accordance with the national guidelines on animal care.

The operative procedure for inducing MI was carried out as previously described [12]. In brief, male Wistar rats (250–320 g, *n* = 48) were intubated, ventilated, and placed in a supine position with paws taped to electrocardiograph (ECG) electrodes. Anesthesia was maintained by continuous inhalation of isoflurane (1.5% v/v; Abbott) in a gas mixture (60% oxygen + 40% air). After disinfecting the thorax skin, the chest was opened with a lateral cut along the left side of the sternum. After identification of the coronary left artery descending artery (LAD), ligation was preceded with a 6–0 polypropylene suture (Prolene, Ethicon) with a tapered needle passed underneath the LAD. The successful occlusion of the LAD was verified visually under the microscope by the absence of blood flow in the epicardium (a pale surface) as well as significant S-T segment elevation. The occlusion was maintained for 90 min to induce a transmural infarction until the suture was released. In all MI experiments, the pericardial sac was carefully sealed with 10–0 silk suture before the chest was closed in layers. Animals were then weaned from ventilation and placed in a warm and oxygen-enriched environment until fully recovered.

### Collection of pericardial fluid and isolation of pericardial adipose-derived stem cells

Pericardial fluid (PF) was sampled from the rats 5 days after MI. Under deep anesthesia with isoflurane, a blood sample was first taken and the thorax was then carefully opened underneath the diaphragm. In some cases, the pericardial sac was gently detached from its partial adherence to the thoracic wall to maintain the integrity of the pericardial sac. The whole pericardial sac together with surrounding tissue including parts of the lung and esophagus was then quickly taken out of the thoracic cavity with forceps. After adsorbing the outside blood, the liquid inside the pericardial cavity (known as PF) was collected into a prechilled falcon tube and centrifugated at −4 °C and 4000 rpm for 5 min. The supernatant was then aliquoted and stored at −80 °C for further analysis.

The injury-associated pericardial adipose-derived stem cells (pADSC) were isolated in the present experiments from the rats 5 days after the induction of MI. In brief, the MI rats were sacrificed with CO_2_ and immersed in 75% ethanol for 3 min for whole-body disinfection. Under sterilized conditions, the chest was opened and pericardial adipose tissue was prepared for either RNA extraction, histology, or isolation of the stromal fraction of adipose tissue. According to a modified protocol reported previously [12], the adipose tissue was washed twice with sterilized phosphate-buffered saline (PBS) and minced into small pieces (about 1×1×1 mm^3^), and subsequently 5 ml of digestion solution containing 84 U/mL of collagenase II (Biochrom, Beijing, China) in PBS was added. The digestion process was maintained at 37 °C for 35 min on a shaker at a rate of 30 spm until the activity of the collagenase was neutralized by the addition of 2 ml fetal calf serum (FCS). The resultant cell suspension was then spun down at 1000 rpm for 5 min to remove the cell debris and fat droplets in the supernatant and the cell pellet was resuspended by gently pipetting with basic medium containing low-sugar Dulbecco’s modified Eagle’s medium (DMEM; Sigma), supplemented with 30% FCS, penicillin (100 U/ml), streptomycin (0.1 mg/ml), and glutamine (2 mM). The cell suspension was seeded at a density of 2 × 10^3^/cm^2^ in a T25 culture flask and incubated at 37 °C with 5% CO_2_. Nonadherent cells were removed 24 h after initial plating by intensely washing the plates, and the resulting adherent stromal fraction was then termed pADSC. The freshly isolated pADSC were cultivated at 37 °C with 5% CO_2_ for about 5–7 days when colony-like aggregates were formed.

### Purification and characterization of WT1-positive pADSC

After further cultivation of pADSC for 3–5 days, small, round, phase-bright cells (PBC) sprouted from the spherical structures over a bed of stromal-like cells. Once they reached 80–90% confluence, the PBC were isolated using 0.01% trypsin-EDTA (Sigma) and: 1) replated on poly-d-lysine-coated T25 culture flask for successive sphere formation; 2) replated on gelatin-coated chamber slides for immunostaining; 3) resuspended for flow cytometric characterization.

For the analysis of the surface epitopes of WT1-positive PBC (WT1^pos^) in relation to attached WT1-negative cells (WT1^neg^, isolated from healthy animals), the following cell surface epitopes were marked with antimurine antibodies: CD29, CD31, CD34, CD44, CD45, CD90, IgG1 (Becton Dickinson), and CD106 (Miltenyi Biotec). In brief, cells were incubated with antibodies at a dilution of 1:100–200 for 10 min at room temperature. After three washes with PBS containing 10% FCS, about 3000 labeled cells were analyzed using a FACScan flow cytometer running CellQuest software (Becton Dickinson).

For the analysis of the expression of nuclear antigens, the PBC were seeded on chamber slides (Ibidi, Germany) and fixed with 1% paraformaldehyde and permeabilized with 0.01% Triton for 30 min. After 1 h of blocking with 5% normal goat serum (NGS), cells were incubated overnight at 4 °C with primary antibodies, including polyclonal anti-WT1 (C-19), isl-1, gata-4, Sox2, Tbx18, and polyclonal anti-ki-67 (1:100, Santa Cruz). After three washes with 1% NGS-PBS buffer, secondary TRTIC-conjugated antibody (goat IgG, 1:100, Santa Cruz) was added and incubated for an additional 60 min at room temperature. Nuclei were counterstained with DAPI (DAKO) and the slides were sealed with Prolong Gold® (Life Science). All visualizations were digitalized using a fluorescent microscopy (Zeiss, Germany) and software (Axiocam, Zeiss) or a phase-contrast microscope using a digital camera (UC30, Olympus). For quantification, the percentage of positively stained cells was counted in relation to the total cells (DAPI-positive) in 10 representative fields from three independent experiments (at 100× or 200× magnification).

### Induction of WT1-expressing PBC toward cardiac lineages

To examine the potential of both WT1^neg^ and WT1^pos^ pADSC towards cardiac differentiation, cells at passage 2 were seeded at a density of 3.0 × 10^3^ cells per cm^2^ onto 0.1% gelatin-coated eight-chamber slides. As soon as subconfluence was reached, the culture medium was switched into DMEM medium supplemented with only 3% FCS + 5% horse serum for cardiac differentiation. The medium was changed every 3 days up to 2 weeks until the cells were fixed and stained immunocytochemically either for cardiac troponin T (cTnT; Santa Cruz) for assessment of myogenic differentiation, or von Willebrand factor (vWF; DAKO) for endothelial differentiation.

### Polymerase chain reaction (PCR) analysis and concentration of cytokines

The pericardial adipose tissue either from the MI or sham-operated rats and cultured pADSC was sampled for total RNA extraction using the RNeasy mini kit (Qiagen, Germany). RNA for reverse transcription-polymerase chain reaction (RT-PCR) was converted to cDNA with a first-strand cDNA synthesis kit (Invitrogen, USA) according to the manufacturer’s recommendations. PCR products were amplified and quantified in a StepOne PCR apparatus (Applied Biosystems). The following primers for Taqman qPCR were used in the present study: WT1, Rn00580566_m1; IGF-1, Rn99999087_m1; VEGF-b, Rn01454585_g1; HGF, Rn00566673_m1; FGF-2, Rn00570809_m1; nkx2.5, Rn00586428_m1; gata4, Rn01530459_m1; isl-1, Rn01494040_m1.

For cytokine profiling of the PF, we measured a panel of cytokines using the Bioplex assay according to the instructions of the manufacturer (Rat cytokine 12-plex; Bio-Rad Shanghai). The concentration of thymus β4 in the culture medium was measured by a commercially available enzyme-linked immunosorbent assay (ELISA) kit (Abcom China) performed according to the manufacturer’s instructions.

### Reparative activity of WT1^pos^ cells in vivo

To further examine the reparative activity of WT1^pos^, we injected cells into the rat hearts after 90 min ischemia/reperfusion as described above. In brief, male Wistar rats (250–320 g, *n* = 13) were randomized into two groups prior to operation, either receiving injection of WT1^pos^ (*n* = 12) cells or the adherent cells which showed minimal expression of WT1 from heathy rats (WT1^neg^, *n* = 11, one animal died from ventricular fibrillation after the induction of cardiac ischemia). After being washed with FCS-free culture medium, a total of one million cells were resuspended in 60 μl PBS and injected directly into three spots at the border zone of the infarcted ventricular free wall over about 2 min. After animals recovered from the transplantation procedure, the chest was closed. Animals were weaned from ventilation and placed in a warm and oxygen-enriched environment until they fully recovered.

Animals were allowed to live in an individual cage until the analysis was performed (5 days after transplantation for apoptotic assays, and 28 days for functional analysis). For the assessment of cardiac contractility function in both groups, echocardiography (Hewlett Packard Sonos 5500 equipped with a 15-MHz linear array probe) was performed. Briefly, the animals were anesthetized by inhalation of isoflurane (2.0% v/v) using a home-made mask and the short axes at the mid-ventricular sections were acquired in M-mode. All measurements were repeated three times in each animal to minimize technical deviations. All data were stored and injection fraction (EF) and fractional shortening (FS) as the key parameters for cardiac function were analyzed offline with customized software (Hewlett Packard).

After all measurements were finished, rats were sacrificed with CO_2_ and the hearts were excised. After washing once in ice-cold PBS, the hearts were carefully positioned and embedded in Tissure-Tek® (Leica, Germany) for histological analysis.

### Immunohistochemistry

Cryosections (8 μm thick) of either pericardial adipose tissue or heart samples (either 5 days or 28 days after transplantation) were made for either staining with hematoxylin and eosin (H&E) or indirect immunostaining. Heart sections were fixed with paraformaldehyde for 10 min at room temperature and immunohistochemical staining was performed in a similar way to the procedures on the chamber slides as described above. The following primary antibodies were used in the present study: WT1 as a reprogram marker, ki-67 as a proliferation marker, cTnT as a cardiac marker or vWF as an endothelial marker (see above), and Caspase 3 (Casp3; Abcam) as the marker for inevitable apoptotic events.

After all the images were acquired, vessel density was manually counted in sections of the vWF immunostaining (red) in at least 10 randomly selected areas (at 200× magnification) within the infarcted area. The percentage of cardiomyocytes was derived from the cardiomyocytes (cTnT-positive, green) in relation to the total tissue section. The total apoptotic cardiomyocytes (cTnT and Casp3 dual positive) were counted throughout the mid-ventricular section. The infarct size was calculated as the percentage of the length of the scar tissue in relation to the ventricular circumference derived from the H&E staining. All analyses were performed using CellSence imaging analysis software.

### Analysis of apoptotic activity in vitro

The protective activity of WT1^pos^ cells against apoptosis was tested on neonatal cardiomyocytes in vitro. Neonatal cardiomyocytes were isolated by enzymatic dissociation of a small mass of cardiac ventricles (1–3 mm^3^) from 1-day to 2-day postnatal Wistar rat neonates. In brief, the ventricular tissue pieces were subjected to multiple rounds of enzymatic digestion by collagenase II (see above). Cells in the digestion solution were repeatedly collected by gravity sedimentation, and then centrifuged at 1000 rpm for 5 min at 4 °C to remove collagenase II solution and then resuspended with DMEM culture medium (Sigma) supplemented with 20% FCS (HyClone), penicillin/streptomycin (100 U/mL), and l-glutamine (1 mmol/L). Subsequently, the cells were differentially replated at a density of 1 million for 60 min on gelatin-coated petri dishes to remove preadherent nonmyocytes.

On the following day, the cells were washed three times with sterile PBS to remove cell debris and the culture medium was changed to the conditioned medium (CM) collected from either the WT1^neg^ or WT1^pos^ cell populations and stressed by the addition of 2 μM H_2_O_2_. Twenty-four hours later, 0.5 μm of fluorescent dye (MitoProbe™ JC-1, Sigma) was added into the culture medium to trace mitochondrial integrity as the onset of cellular apoptosis. The dishes were maintained in a dark environment for 45 min and JC-1 green (monomers) and red fluorescence (aggregates) were visualized using a fluorescent microscope (Zeiss, Germany). The electronic integrity (Δψm) of the mitochondrial membrane was indicated by the ratio of red/green fluorochrome intensity measured by CellSence imaging analysis software and compared within the groups including H_2_O_2_ control, CM from either WT1^neg^ or WT1^pos^ cells, and supplements of either monoclonal antibody against HGF (100 ng/ml; H170, Santa Cruz) to the wells with WT1^pos^ CM (WT1^pos^ + AB), or HGF (20 ng/ml; JKChem, Shanghai) to the wells with WT1^neg^ CM (WT1^neg^ + HGF).

### Statistical analysis

Data are presented as the mean ± standard deviation. A student *t* test with Welch’s correction was applied to compare WT1 expression and the reparative activity of WT1^pos^ with the WT1^neg^ group. The intensity ratio of red/green fluorochromes in the apoptotic experiment was compared with one-way analysis of variance (ANOVA). Differences were considered significant at *p* < 0.05. The Prism software package (version 7.0) was used for the statistical analyses.

## Results

### Reactivation of WT1 expression in pericardial adipose tissue after ischemic injury

**Fig. 1:**
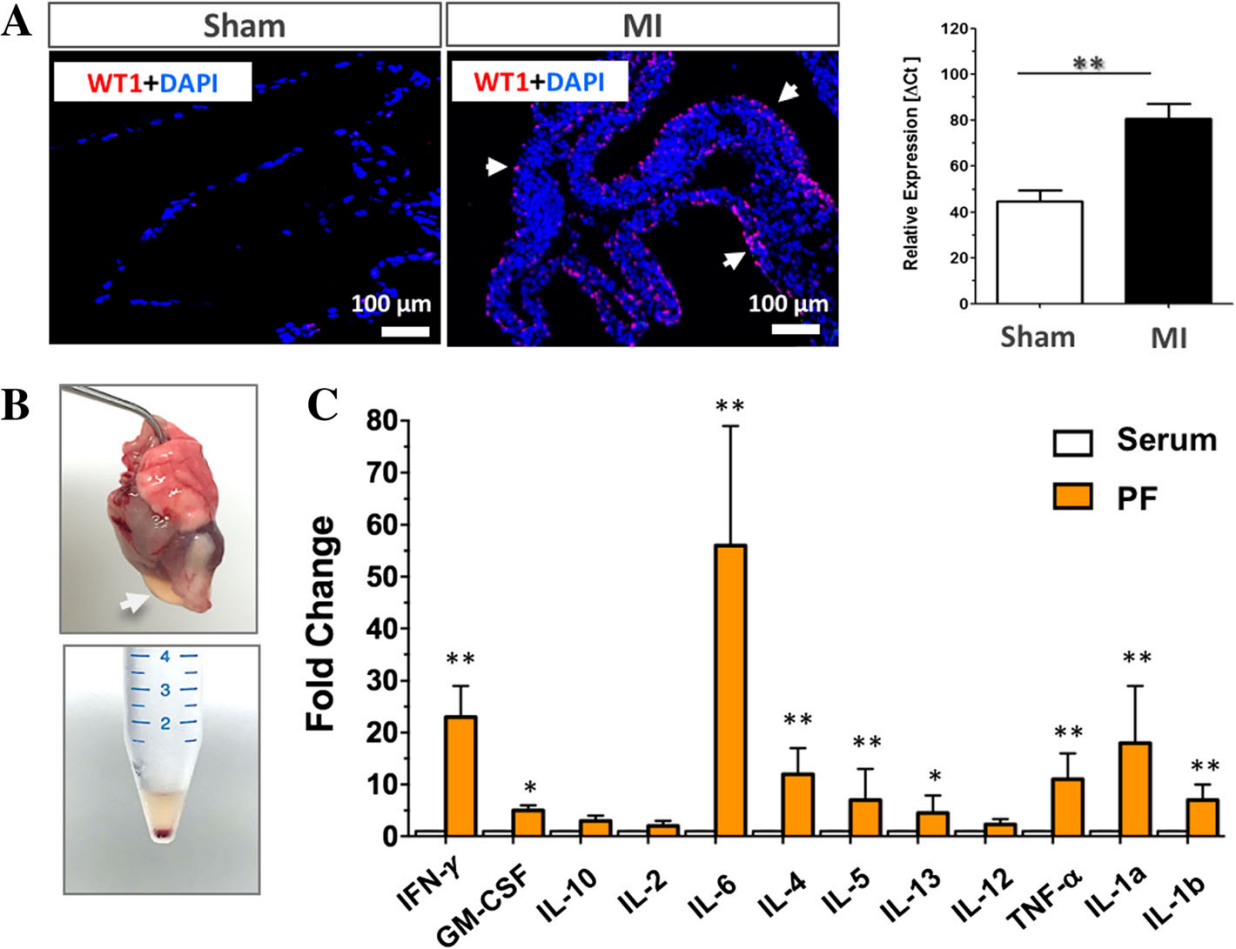
Injury induced re-expression of WT1 in the pericardial adipose tissue. The pericardium, in response to myocardial infarction (MI), expanded in thickness and recapitulated the expression of Wilms’ tumor factor 1 (WT1), locating mainly in the cells of the inner and outer layers of the pericardial tissue (**a**). In an intact model of the pericardial sac, the cardiac transudate (pericardial fluid (PF)) was collected from the MI rats (5 days) (**b**). Bioplex analysis revealed that a panel of cytokines in PF were massively increased in comparison with serum concentration (**c**). **p < 0.01. GM-CSF granulocyte/macrophage colony-stimulating factor, IFN interferon, IL interleukin, TNF tumor necrosis factor

During tissue preparation for the isolation of pADSC from the MI rats, we found that the pericardial adipose tissue was located mainly in the anterior part of the fibrous layer of the pericardium and, sometimes, adhered to the heart surface after ischemic injury. Histological analysis of 5-day MI hearts revealed that the pericardial tissue expanded into a multicellular layer that was composed of multiple ki-67-positive cells, indicating active proliferation of pericardial cells in response to cardiac injury (Fig. 1a). Interestingly, immunofluorescent staining demonstrated an expression of WT1 in a proportion of the pericardial cells of the injured rats, located mainly in the inner and outer parts of the expanded pericardial layers, while this was almost undetectable in the sham animals (1.8% ± 1.0% vs. 18.5% ± 1.2%; *n* = 4, *p* < 0.01; Fig. 1a). The transcriptional level of WT1 mRNA from the whole-tissue homogenate showed the same trend (*n* = 4 and 3, *p* < 0.01; Fig. 1a). WT1 is known as an important transcriptional regulator that is crucial to the embryonic formation of the second heart field and is reactivated in response to cardiac injury, mainly in the dormant epicardial progenitors. Pericardial tissue likely undergoes a similar reactivating process triggered by injury-associated signaling presenting in the PF. In the context, we collected cardiac transudate in a closely sealed pericardial sac 5 days after the induction of MI. The PF was composed of minor blood cells (hematocrit = 3.8% ± 2.2%, *n* = 6; Fig. 1b), indicating slight hemorrhage during the formation of PF from the injured heart, and contained identical protein content to blood serum (data not shown). Analysis of cytokine concentration by Bioplex in PF revealed a panel of inflammatory mediators being significantly abundant in the PF in comparison with the serum concentration in the same rats. Among them, interleukin (IL)-6, IL-4, interferon (IFN)-γ, and tumor necrosis factor (TNF)-α were mostly pronounced (Fig. 1c). Therefore, these results provide direct evidence that the presence of injury-associated signals in the PF may act as important transmitters to reactivate the expression of WT1 in the pericardial adipose tissue.

### Isolation of WT1-expressing cells

**Fig. 2:**
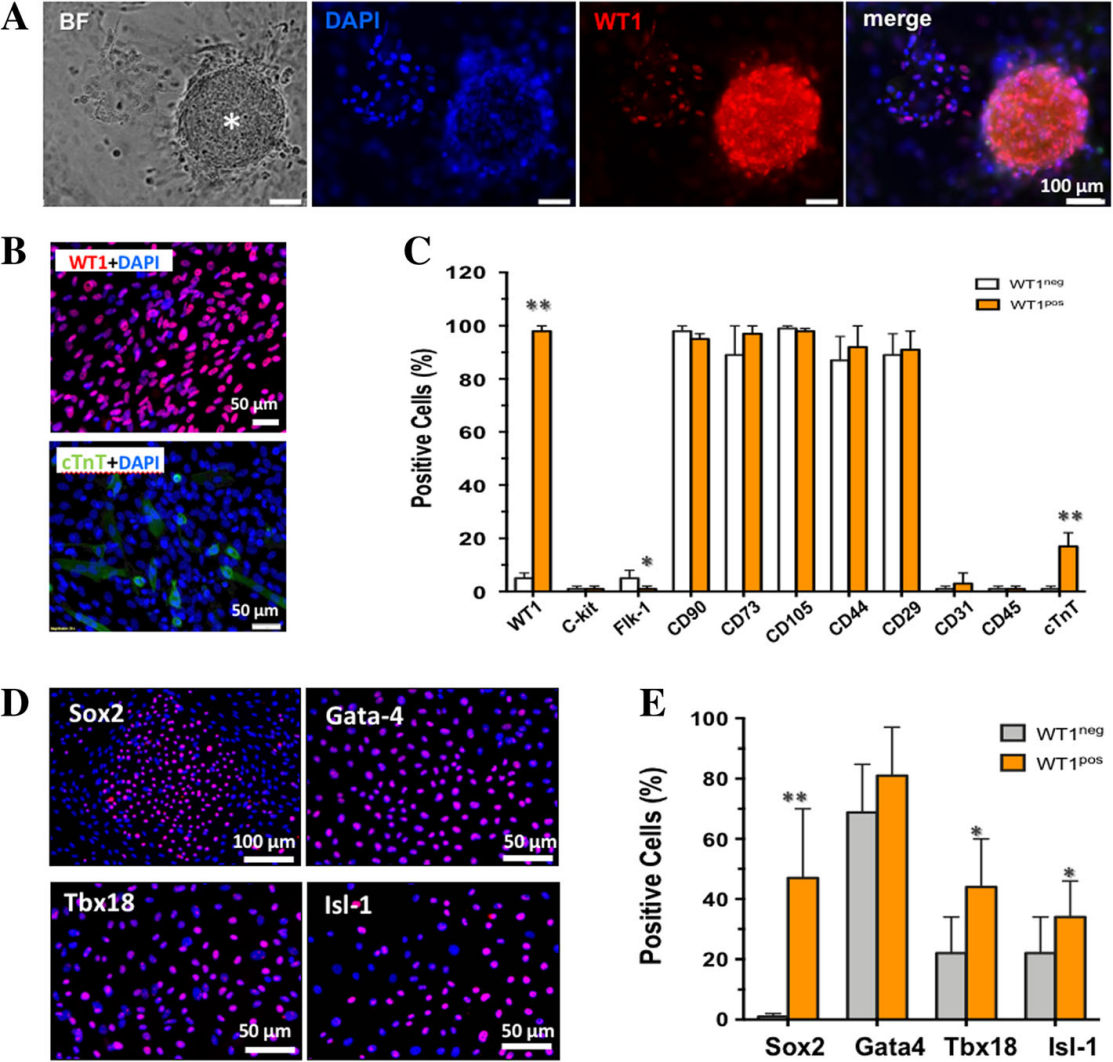
Isolation and characterization of WT1^pos^ pADSC. The Wilms’ tumor factor 1 (WT1)-positive cells in culture formed colony-like structure (asterisk in **a**) and subsequently generated phase-bright cells (PBC) that homogenously express WT1 (WT1^pos^) and partially express cardiac troponin T (cTnT) (**b**). Flow cytometry showed that, although the WT1^pos^ cells shared the canonical surface markers of pADSC as defined in our previous report [12], an increased proportion of cTnT-positive cells was detected (**c**). Furthermore, the WT1^pos^ cells exhibited upregulated expression of the embryonic gene Sox2 and cardiac related genes isl-1and Tbx18 in comparison with WT1^neg^ cells (**d** and **e**). *p < 0.05, **p < 0.01. BF bright field

By enzymatic digestion and cultivation in standard DMEM medium, pADSC formed colony-like aggregates assembled to cardiospheres (Fig. 2a) with a mainly cellular component of WT1-expressing cells. When the culture period was extended for an additional 3–5 days, multiple small, round-shaped, phase-bright cells (PBC) sprouted out of the spheres and loosely attached to the petri dish. Notably, the progeny of spheres after passaging still preserved the expression of WT1 and yielded a relatively homogenous WT1-expressing PBC (WT1^pos^, 98.4% ± 1.2%, *n* = 6; Fig. 2b, c), indicating the sustained expression of WT1 in a subpopulation of cultivated pADSC. Flow cytometric analysis further demonstrated that the WT1^pos^ cells were phenotypically identical to WT1-negative cells (WT1^neg^, from the healthy rats) in the context of CD29, CD44, CD90, and CD105 expression, but were eventually c-kit, CD31, CD34, and CD45 negative (Fig. 2c, *n* = 4). Interestingly, a small fraction of WT1^pos^ cells already showed cardiac commitment by expressing cTnT (18% ± 4.5%; Fig. 2b, c). WT1^pos^ cells were eventually flk-1 negative, indicating that they were phenotypically distinct subset from pADSC that we have previously reported (Fig. 2c) [13]. Given the cardiac potential of WT1^pos^ cells, we further compared a panel of important transcriptional factors that regulate myogenic differentiation. WT1^pos^ cells exhibited a significant upregulation of Sox2, Gata4, Tbx18, and Isl-1 in comparison with WT1^neg^ cells (Fig. 2d, e). Expression of the transcriptional factors at the mRNA level was also confirmed by RT-PCR (data not shown). Therefore, in our experiments by taking advantage of the cellular property of WT1^pos^ cells (semiattachment), we were able to isolate relatively pure WT1-expressing cells from the cultured pADSC and further demonstrated their myogenic potential characterized by their upregulation of cardiac transcriptional factors and, in a small proportion, cardiac commitment under default conditions.

### Differentiation potential of WT1^pos^ cells into cardiac lineages

**Fig. 3:**
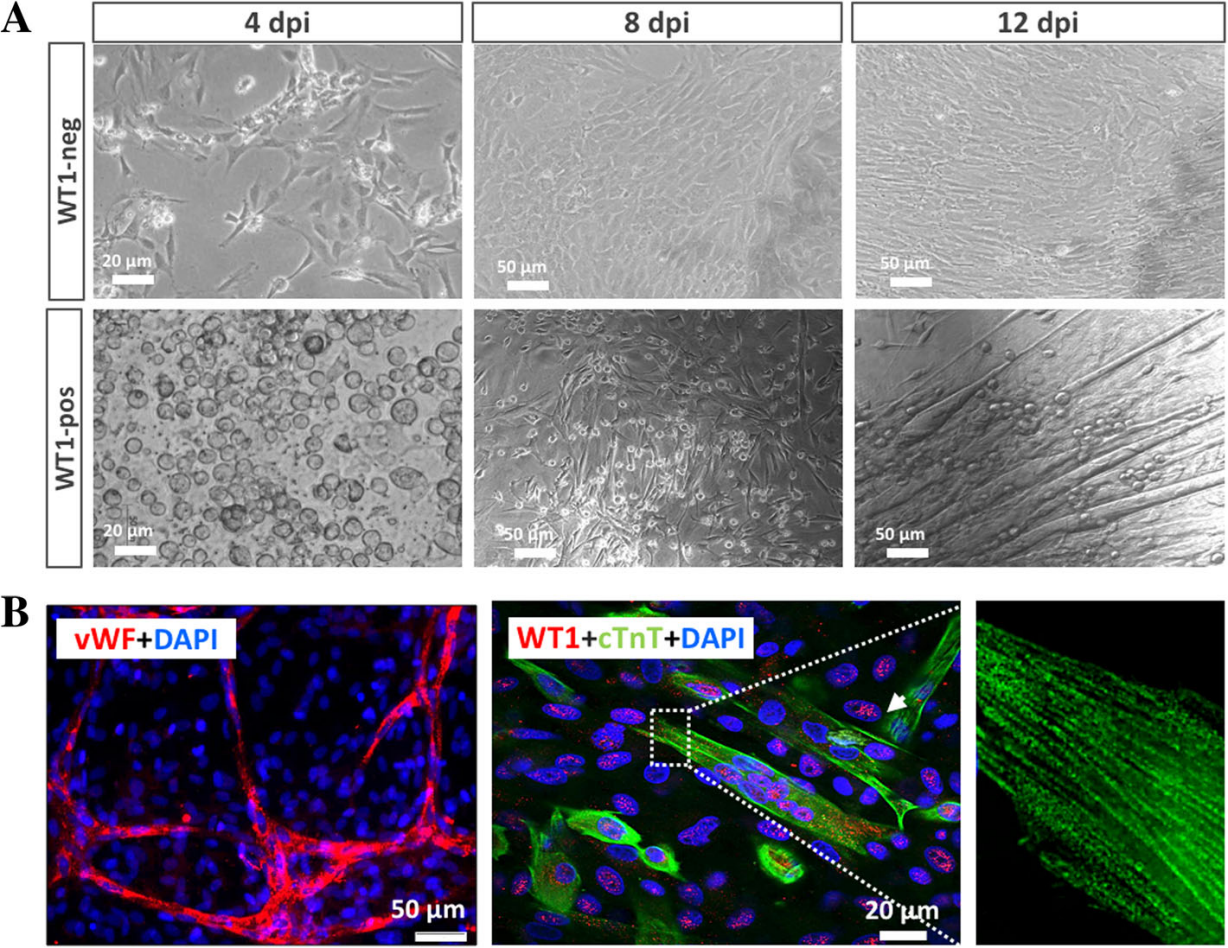
Differentiation potential of Wilms’ tumor factor 1 (WT1)-positive (WT1^pos^) cells into cardiac lineages. The WT1^pos^ cells (WT1-pos) showed small and round morphology (**a**) and, when induced under defined conditions (3% FCS + 5% horse serum) on gelatin-coated dishes, they became elongated and formed giant cells 12 days after induction (dpi) and developed spontaneously contracting activity in the dish. The morphometric changes were not observed in WT1^neg^ populations (WT1-neg) under identical culture conditions. Immunocytochemistry further revealed the formation of ramified vessel-like structures with the expression of the endothelial marker von Willebrand factor (vWF) and myogenic lineage expressing cardiac troponin T (cTnT) that formed striation structures (**b**)

We then systematically assessed the differentiation potential of WT1^pos^ cells. In contrast to a differential protocol previously used [12], we showed here that a mere reduction of FCS concentration from 30% to 3% and a supplement with 5% horse serum readily induced WT1^pos^ cells into cardiac differentiation. After 8 days of cultivation, the attached WT1^pos^ cells elongated in size and exhibited a spindle-like morphology, and subsequently formed giant cells (Fig. 3a). The unique morphometric changes, however, were not observed in the WT1^neg^ population (Fig. 3a). Immunocytochemical analysis revealed that, while the WT1^neg^ cells showed almost negative staining for vWF, an endothelial marker, and cardiac cTnT (data not shown), the WT1^pos^ cells were able to form ramified vessel-like structures that stained positive for vWF at an efficiency of 28% (Fig. 3b, *n* = 4), and differentiated into the myogenic lineage at an efficiency of 22.3% (Fig. 3b, *n* = 6). The elongated cells showed a striation pattern and gained spontaneously contracting activity in culture, indicating maturation of cardiac-committed WT1^pos^ cells. Therefore, WT1^pos^ cells bear an enhanced competency of cardiac potential in vitro and, thus, represent attractive injury-induced cardiac progenitors genearting from pericardial adipose tissue.

### Reconstitution of the injured heart in vivo

**Fig. 4:**
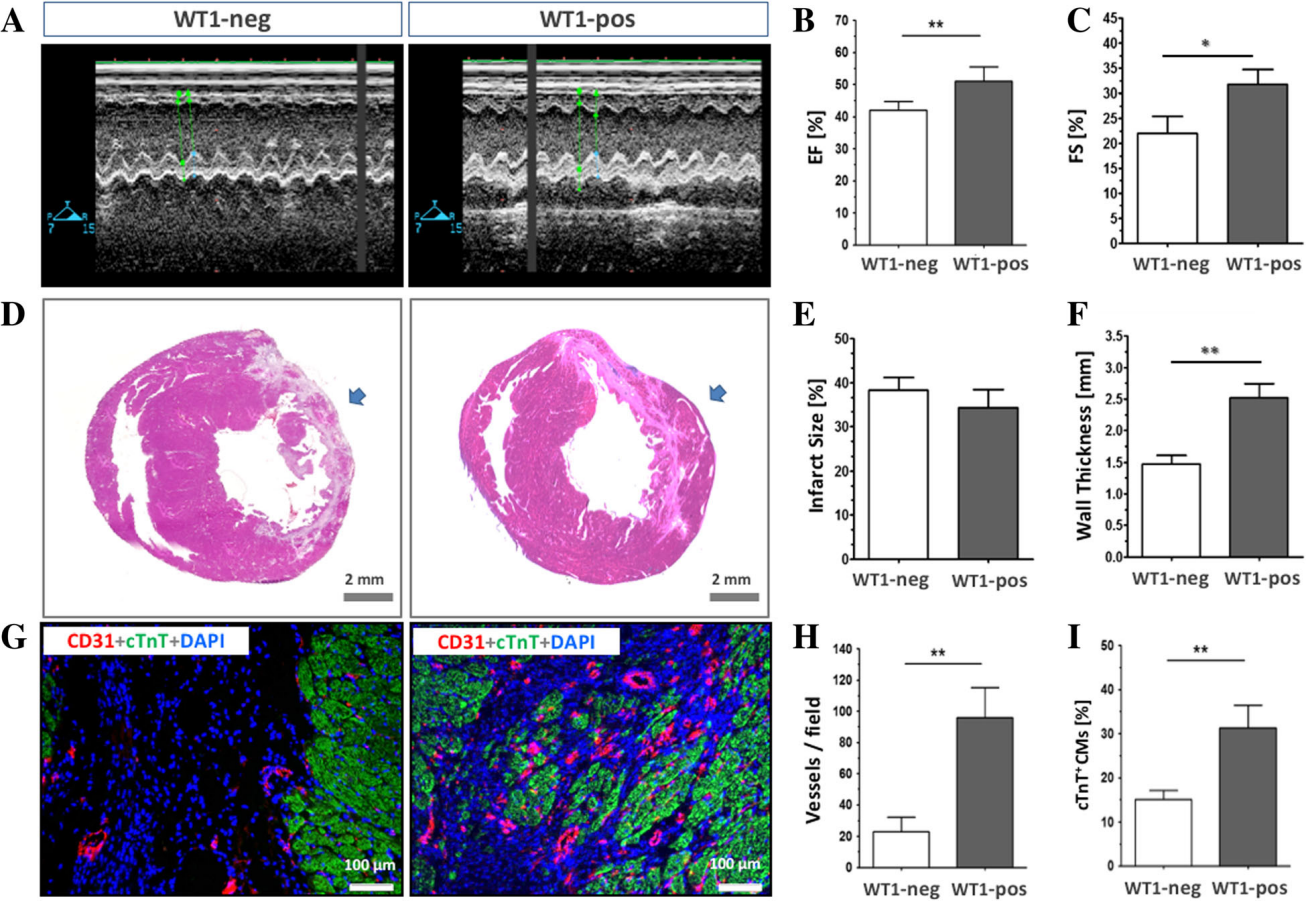
Cardiac repair of Wilms’ tumor factor 1 (WT1)-positive (WT1^pos^) cells after in-vivo transplantation. The WT1-positive cells (WT1-pos) were directly injected into the infarcted myocardium and the cardiac function and histology were assessed after 28-day follow-up. The WT1^pos^ cell-treated heart showed improved cardiac ejection fraction (EF) and fractional shortening (FS) in comparison with WT1-negative controls (WT1-neg) assessed by echocardiography (**a**–**c**). Remarkably, although the infarcted size was not altered (**e**), significantly more cardiomyocytes (CMs; cardiac troponin T (cTnT)-positive; **g** and **i**) and vessels (vWF-positive; **g** and **h**) were formed, and thus the anterior wall was thickened (**f**) at the site of injection (arrows in **d**), indicating the augmented myogenic and angiogenic effects induced by cell transplantation. *p < 0.05, **p < 0.01

To examine the reparative activity in vivo, we intramyocardially injected one million WT1^pos^ cells into three spots within the infarcted area in an experimental MI model in rats. After 28 days we evaluated the structural reconstitution and functional improvement in WT1^pos^ cell-treated animals in comparison with controls treated with WT1^neg^ cells. As shown in Fig. 4a–c, echocardiographic analysis demonstrated that the cardiac ejection fraction (EF) and fractional shortening (FS), two important indicators of global contractile function of the heart, were significantly improved in the WT1^pos^ cell-treated animals (EF: 42.06% ± 3.3% vs. 52.02% ± 4.1%, *p* < 0.01; FS: 22.00% ± 1.7% vs. 31.75% ± 1.5%, *p* < 0.01; *n* = 5–6) compared with WT1^neg^ cell-treated animals. Histological analysis further revealed that the infarcted ventricular wall eventually turned out to be a thin fibrotic layer lacking in cardiomyocytes (red in H&E staining in Fig. 4d) in the WT1^neg^ cell-treated rats but was significantly thickened in the myocardium after injection of WT1^pos^ cells, mainly in the area of injection (arrow in Fig. 4d). Morphometric analysis showed that, although the infarct size was identical in both groups (Fig. 4e), the thickness of the infarcted wall after WT1^pos^ cell treatment increased by 71.2% compared with WT1^neg^ cell-treated animals (*n* = 4–5, *p* < 0.01). We further explored the cellular constituents responsible for the thickening of the anterior wall using specific antibodies that bind to either mature cardiomyocytes (cTnT for myogenesis) or endothelial cells (vWF for angiogenesis). The immunostaining of heart sections revealed a robust quantity of cardiomyocytes (green in Fig. 4g) as well as vasculature (red in Fig. 4g) in the WT1^pos^ cell-treated hearts in comparison with WT1^neg^ cell-treated hearts (23.00 ± 13.1 vessels / field vs. 96.00 ± 17.7 vessels / field, *n* = 4–5, *p* < 0.01 in Fig. 4h and 15.08% ± 3.1% vs. 31.26% ± 7.7%, *n* = 4–5, *p* < 0.01 in Fig. 4i). These results indicate that the WT1^pos^ cells provide a more pronounced reparative activity compared with WT1^neg^ cells by enhancing both myogenic and angiogenic properties in the injured hearts.

### Trophic factors and antiapoptotic effects

**Fig. 5:**
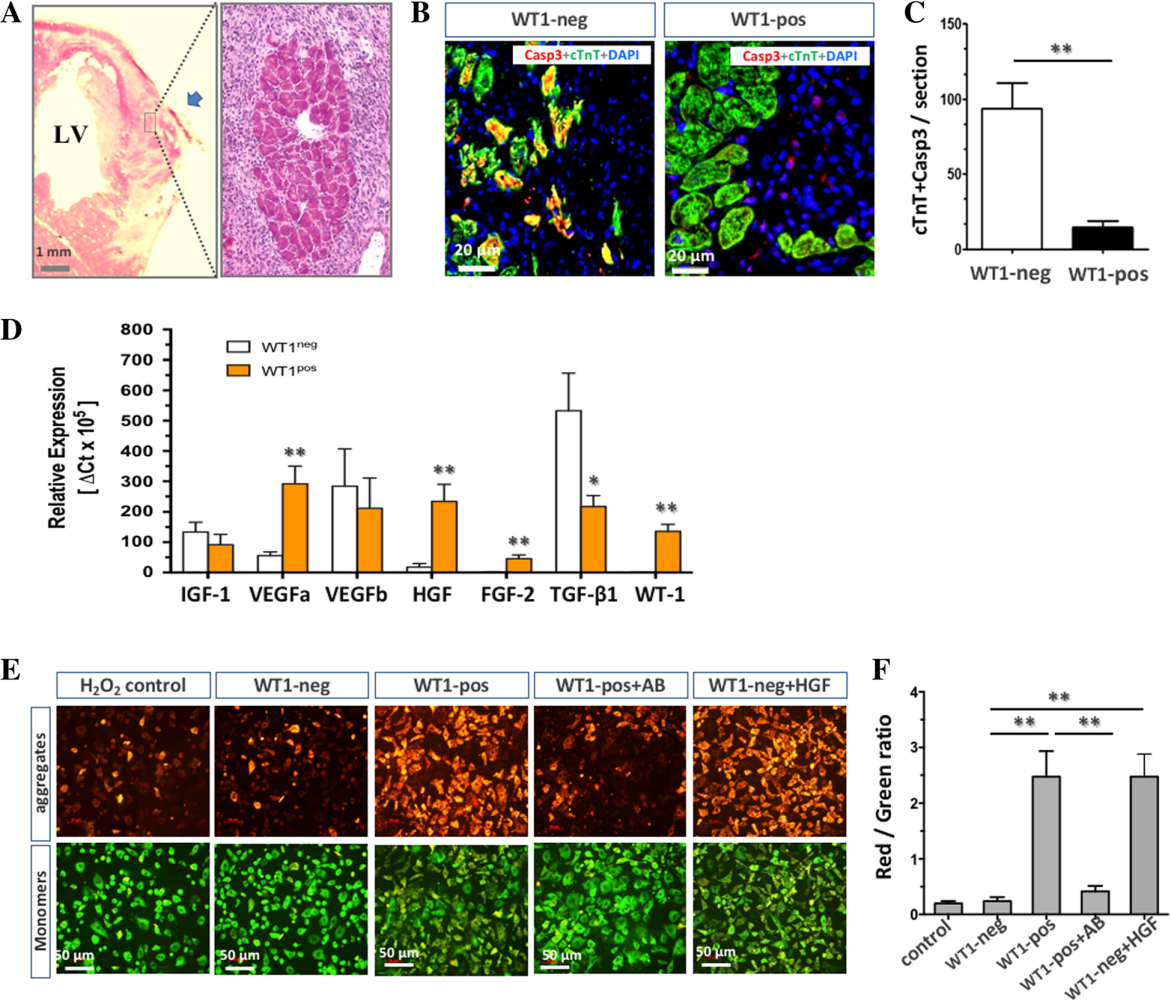
Identification of hepatocyte growth factor as an antiapoptotic mediator. Multiple surviving clusters of cardiomyocytes were found within the infarcted area after receiving Wilms’ tumor factor 1 (WT1)-positive (WT1^pos^) cell injection (WT1-pos) (**a**). These cardiomyocytes showed a lower prevalence of apoptotic events evidenced by caspase 3 (Casp3) staining compared with the WT1^neg^ cell-injected hearts (WT1-neg) (**b**, **c**). Transcriptional analysis revealed a significant upregulation of hepatocyte growth factor (HGF) and vascular endothelial growth factor (VEGF)a in WT1^pos^ cells, whereas insulin-like growth factor (IGF)-1, VEGF-b, and fibroblast growth factor (FGF)-2 remained unchanged (**d**). The conditioned medium from the WT1^pos^ cells prevented the H_2_O_2_-induced apoptosis in neonatal cardiomyocytes, evidenced by the reduced transition of aggregates (red) to monomers (green) of JC-1 dye (**e**, **f**), which was blocked by the addition of HGF antibody (+AB) and was mimicked by the addition of recombinant HGF protein (+HGF). Arrow in (**a**) indicates the site of injection. **p < 0.01. TGF transforming growth factor

We further looked at the intrinsic mechanism by which WT1^pos^ cells induce cardiac repair. In the early stages, at 5 days after transplantation, we found multiple clusters of cardiomyocytes in close proximity to the injection spots within the necrotic myocardium (arrow in Fig. 5a), indicating that injection of WT1^pos^ cells may cause a cardiac protective effect against an ischemic insult. This notion was further supported by the results of immunochemical staining of Casp3 (Fig. 5b) showing that the cardiomyocytes in the WT1^pos^ cell-injected hearts became highly resistant to ischemic stress, while in the WT1^neg^ cell-injected hearts abundant cardiomyocytes inevitably entered an apoptotic process (Casp3-positive; Fig. 5c, *p* < 0.01, *n* = 3).

In an attempt to identify the potential factors secreted by WT1^pos^ cells that mediated the antiapoptotic effects, we analyzed the expression of a panel of trophic factors that have been previously reported to help the beneficial effects of stem cell-based therapy using a Taqman PCR technique. Among those factors tested, vascular endothelial growth factor (VEGF)-a and HGF were found to be increased in the population of WT1^pos^ cells, whereas insulin-like growth factor (IGF)-1, VEGF-b, transforming growth factor (TGF)-β, and fibroblast growth factor (FGF)-2 showed slight or no upregulation in comparison with WT1^neg^ cells (Fig. 5d). Finally, we narrowed it down to HGF as an important mediator for cardiac protection. In culture, we utilized JC-1 as an apoptotic tracer to examine the cardiac protective effects of conditioned medium (CM) on stressed neonatal cardiomyocytes in vitro. The chemical formation of JC-1 monomers caused by oxidative stress of H_2_O_2_ (green in Fig. 5e; *n* = 6) was not affected by WT1^neg^ CM, but was remarkably prevented by WT1^pos^ CM, suggesting the existence of an antiapoptotic effect for WT1^pos^ CM. Notably, this effect was antagonized by the addition of HGF antibody in the WT1^pos^ CM (Fig. 5f; *p* < 0.01, *n* = 5). Furthermore, the protective effects were able to be mimicked by a supplement of recombinant HGF protein in the WT1^neg^ CM (Fig. 5e, f; *n* = 4). Therefore, our in-vitro results revealed HGF as a key WT1^pos^ cell-derived antiapoptotic factor that protects cardiomyocytes from oxidative stress, which likely accounts for the structural and functional benefits yielded by WT1^pos^ cell transplantation in vivo.

## Discussion

The present study demonstrates for the first time that pADSC, in response to injury-induced signaling after MI, recapitulated the expression of WT1 as a hallmark of fetal reprogramming which imparts not only enhanced cellular “stemness” but also was instrumental in promoting cardiac multilineage potential. The injury-“conditioned” pADSC foster cardiac reparative activity by paracrine-mediated angiogenesis and antiapoptosis in cardiomyocytes, exemplifying a paradigm of injury-induced reparative activity that supports tissue homeostasis.

In our previous experiments characterizing the reparative activity of pADSC the pericardial tissue samples were also sometimes taken from MI rats [12, 13] and we found, unexpectedly, that the pADSC isolated from the MI rats exhibited significantly enhanced reparative properties in comparison with the cells from healthy animals. We therefore compared the phenotypic markers of pADSC from two types of animals, in other words healthy and MI rats. Indeed, the pADSC from either healthy or MI rats showed identical expressions of several key makers for mesenchymal stem cells (Fig. 2c). Given that tissue injury may rapidly shift the quiescent stem cells into an activated state unique to regeneration [14], we reasoned that the injury-“conditioned” pADSC after MI may readily acquire certain activities preferential for cardiac repair.

In injured tissue, the production of danger signals known as damage-associated molecular patterns (DAMPs) from cells stressed, damaged, and/or dying in the local tissue creates a unique inflammatory environment that, mostly via the release of cytokines [19], rapidly shifts the quiescent progenitors into activated, transient states to meet the demands for injury-induced repair [20, 21]. This situation is reminiscent of regenerating muscle, in which renewed satellite cells retain both their stemness and multipotency and are also known to arise from a heterogeneous pool of activated stem cells [22]. In the adult heart, the dormant epicardial progenitors, mainly through MI-induced release of thymus β4 [23], recapitulated the expression of one of the important embryonic transcriptional factors, WT1, that fosters cardiac repair by cellular replacement [9] or in a paracrine manner [17]. WT1 was known as a tumor repressor gene causatively involved in eponymous nephroblastoma, but was recently revealed as a transcription factor with strong transactivating potential in organogenesis [24]. In the adult heart, the re-expression of WT1 in the epicardial progenitor cells is typically considered as a hallmark of cellular reprogramming analogous to its developmental program [8, 9].

Although the chemical nature of the stimulatory molecules that orchestrate a series of cellular events of fetal reprograming remain unclear, several studies have suggested that factors in the PF formed after MI were critical to the reactivation process in epicardial cells [18], but also in pericardial cells that, in a similar scenario, have been exposed to an inflammatory environment such as PF. Here, we have developed a rat model with an intact pericardial sac in which the cardiac transudate was accumulated and could be sampled for biological assays; this enabled us to analyze the bioactive components that triggered the reprogramming process in both epicardial and pericardial cells [7]. We have demonstrated the formation of PF in the pericardial sac after cardiac injury with a massive release of proinflammatory cytokines and IL-6 that may act as important signals to initiate pADSC into a reparative form by the expression of WT1. In contrast with a previous publication [23], we failed to confirm the role of thymosin β4 in epicardial/pericardial reprograming since thymosin β4 was found to be under its detectable level in PF. This rather negative observation may have resulted from the insensitivity of the ELISA kit that we used in the present experiments and, probably, from a short half-life period of this peptide within PF that accumulated for as long as 5 days. Nevertheless, we cannot rule out the contribution of thymosin β4 in the induction of WT1 expression on the basis of less abundance; it may act more potently and effectively. Interestingly, we found the release of a panel of cytokines was massively increased and seemed to be crucial in the induction of fetal reprograming in pADSC [25]; however, the individual factors in the inductive process need to be further characterized.

The pericardium contains heterogeneous populations that react differently to injury-associated signaling [26]; the cells residing in the outer and inner layer of the expanded pericardium mainly recapitulated the expression of WT1 (Fig. 1a). Given that WT1 regulates the important procedure of epithelial-to-mesenchymal transition (EMT) [27], it is possible that some pADSC underwent an injury-induced EMT and acquired the biological properties of mesenchymal stem cells. Indeed, our in-vitro experimental results demonstrated that the WT1^pos^ cells gave rise to successive formations of cardiospheres (Fig. 2a–c) similar to cardiac stem cells [28], and boosted cardiac-related genes in comparison with the WT1^neg^ cells. Most interestingly, WT1^pos^, but not WT1^neg^ cells, without any chemical induction, differentiated into cardiac lineages only by reduction of FCS concentration in the culture medium (Fig. 3), suggesting the enhanced cardiac potential in the injury-“conditioned” pADSC [29].

The striking differentiation potential of WT1^pos^ cells led us to examine their therapeutic application in the treatment of ischemic heart disease. Transplantation of WT1^pos^ cells into the damaged heart prevented structural and functional deterioration after myocardial infarction by enhancing both angiogenesis and myogenesis within the necrotic myocardium (Fig. 4). The beneficial effects observed in the present experiment were more pronounced than previously reported results either using the unfractionated population [12] or the flk-1-positive pADSC tested in our laboratory [13]. Since WT1^pos^ cells likewise failed to adapt to long-term engraftment in the host myocardium (data not shown), the reparative activity can be most probably attributed to a paracrine effect rather than direct formation of de novo cardiomyocytes [12]. In the present experimental setting, we found multiple surviving clusters of cardiomyocytes that showed high resistance to apoptosis (Casp3-positive) after WT1^pos^ cell injection. This result pinpoints a unique activity of WT1^pos^ cells that may stabilize vasculatures and preserve the damaged cardiomyocytes by an antiapoptotic action.

Adipose stem cells are a source of beneficial factors and/or exosomes for tissue repair [30]. In an attempt to identify the protective factors, we compared the expression of a panel of trophic factors that were previously reported to induce cardiac repair in WT1^pos^ and WT1^neg^ cells [31], and found that HGF is the only one strikingly upregulated in WT1^pos^ cells. This result led us to further define HGF as a pivotal factor that mediates the antiapoptotic activity of WT1^pos^ cells in vitro (Fig. 5e, f). HGF may exert a broad spectrum of protective actions through the activation of phosphoinositide 3-kinase (PI3K)/Akt and mitogen-activated protein kinase (MAPK) cascades [32] and the regulation of the cardiac immune response after injury [33]. Therefore, HGF functions not only as a regulator for the maintenance of normal cardiac function in adults [34], but also as an important angiogenic factor [35] and chemoattractant for cardiac progenitors [36] in the course of cardiac repair. Several studies using HGF-based gene therapy as well as recombinant proteins have shown that HGF ameliorated cardiac function in failing hearts through its proangiogenic, anti-inflammatory, antifibrotic, and antiapoptotic activities [35, 37–39]. In the present experiments, we confirmed HGF, likely cooperating with VEGFa, as an important paracrine factor that enhances therapeutic angiogenesis and promotes the survival of cardiomyocytes under in-vivo and in-vitro conditions.

The molecular link between WT1 expression and upregulation of HGF remains unknown. WT1 is not only associated with tumorigenesis but also functions as a master switch in the development of vasculature in multiple organs [40, 41]. WT1 was also found to be reactivated in adults in response to injury-associated signals in the epicardial cells and to play a key role in cardiac repair by de novo formation of cardiomyocytes [9, 29] as well as by secreting angiogenic factors [17]. The same procedure likely also occurred in pericardial adipose stem cells that are subject to the injury-associated signals presenting in pericardial fluid [18]. The activated pADSC with WT1 expression were found to yield much more pronounced cardiac protective effects than WT1^neg^ cells, likely via the enhanced secretion of HGF. Future experiments using molecular biology approaches to reveal the transcriptional regulation of HGF by WT1 may provide us a rational understanding of how partial reprogramming in certain type of cells significantly evokes their reparative activity [42].

## Conclusion

In conclusion, the present study demonstrated that injury-associated signaling trigged re-expression of the WT1 transcript in pADSC and potentiated cardiac differentiation as well as the activity of cardiac repair by enhancing HGF production. In-vivo transplantation of WT1^pos^ cells into the infracted heart yielded significant cardiac benefits through HGF-mediated proangiogenic and antiapoptotic effects. As such, WT1^pos^ cells may represent a useful autologous cell donor from infarcted patients in cell-based therapy.

## Abbreviations

Casp3: Caspase 3
CM: Conditioned medium
cTnT: Cardiac troponin T
DMEM: Dulbecco’s modified Eagle’s medium
EDPC: Epicardial-derived progenitor cells
EF: Ejection fraction
EMT: Epithelial-to-mesenchymal transition
FCS: Fetal calf serum
FS: Fractional shortening
H&E: Hematoxylin and eosin
HGF: Hepatocyte growth factor
IL: Interleukin
LAD: Left artery descending artery
MI: Myocardial infarction
NGS: Normal goat serum
pADSC: Pericardial adipose-derived stem cells
PBC: Phase-bright cells
PBS: Phosphate-buffered saline
PCR: Polymerase chain reaction
PF: Pericardial fluid
RT-PCR: Reverse transcription-polymerase chain reaction
VEGF: Vascular endothelial growth factor
vWF: Von Willebrand factor
WT1: Wilms’ tumor factor 1
WT1^neg^: WT1 negative pADSC
WT1^pos^: WT1 positive pADSC

## References

[1] Jopling C, Sleep E, Raya M, Martí M, Raya A, Belmonte JCI (2010). Zebrafish heart regeneration occurs by cardiomyocyte dedifferentiation and proliferation. Nature.

[2] Anversa P, Kajstura J, Leri A, Bolli R (2006). Life and death of cardiac stem cells: a paradigm shift in cardiac biology. Circulation.

[3] Leri A, Rota M, Pasqualini FS, Goichberg P, Anversa P (2015). Origin of cardiomyocytes in the adult heart. Circ Res.

[4] Ptaszek LM, Mansour M, Ruskin JN, Chien KR (2012). Towards regenerative therapy for cardiac disease. Lancet.

[5] Eschenhagen T, Bolli R, Braun T, Field LJ, Fleischmann BK, Frisén J (2017). Cardiomyocyte regeneration: a consensus statement. Circulation.

[6] Smart N, Riley PR (2011). The epicardium as a candidate for heart regeneration. Futur Cardiol.

[7] Limana F, Capogrossi MC, Germani A (2011). The epicardium in cardiac repair: from the stem cell view. Pharmacol Ther.

[8] van Wijk B, Gunst QD, Moorman AFM, van den Hoff MJB (2012). Cardiac regeneration from activated epicardium. PLoS One.

[9] Smart N, Bollini S, Dubé KN, Vieira JM, Zhou B, Davidson S (2011). De novo cardiomyocytes from within the activated adult heart after injury. Nature.

[10] Winter EM, Grauss RW, Hogers B, van Tuyn J, van der Geest R, Lie-Venema H (2007). Preservation of left ventricular function and attenuation of remodeling after transplantation of human epicardium-derived cells into the infarcted mouse heart. Circulation.

[11] Chong JJH, Chandrakanthan V, Xaymardan M, Asli NS, Li J, Ahmed I (2011). Adult cardiac-resident MSC-like stem cells with a proepicardial origin. Cell Stem Cell.

[12] Wang X, Zhang H, Nie L, Xu L, Chen M, Ding Z (2014). Myogenic differentiation and reparative activity of stromal cells derived from pericardial adipose in comparison to subcutaneous origin. Stem Cell Res Ther.

[13] Wang X, Liu X, Zhang H, Nie L, Chen M, Ding Z (2016). Reconstitute the damaged heart via the dual reparative roles of pericardial adipose-derived flk-1+ stem cells. Int J Cardiol.

[14] Lin B, Coleman JH, Peterson JN, Zunitch MJ, Jang W, Herrick DB (2017). Injury induces endogenous reprogramming and dedifferentiation of neuronal progenitors to multipotency. Cell Stem Cell.

[15] Cheng B, Chen HC, Chou IW, Tang TWH, Hsieh PCH (2017). Harnessing the early post-injury inflammatory responses for cardiac regeneration. J Biomed Sci.

[16] Gadye L, Das D, Sanchez MA, Street K, Baudhuin A, Wagner A (2017). Injury activates transient olfactory stem cell states with diverse lineage capacities. Cell Stem Cell.

[17] Zhou B, Honor LB, He H, Ma Q, Oh J-H, Butterfield C (2011). Adult mouse epicardium modulates myocardial injury by secreting paracrine factors. J Clin Invest.

[18] Limana F, Bertolami C, Mangoni A, Di Carlo A, Avitabile D, Mocini D (2010). Myocardial infarction induces embryonic reprogramming of epicardial c-kit+ cells: role of the pericardial fluid. J Mol Cell Cardiol.

[19] Kono H, Rock KL (2008). How dying cells alert the immune system to danger. Nat Rev Immunol.

[20] Karin M, Clevers H (2016). Reparative inflammation takes charge of tissue regeneration. Nature.

[21] Godwin JW, Pinto AR, Rosenthal NA (2017). Chasing the recipe for a pro-regenerative immune system. Semin Cell Dev Biol.

[22] Collins CA, Olsen I, Zammit PS, Heslop L, Petrie A, Partridge TA (2005). Stem cell function, self-renewal, and behavioral heterogeneity of cells from the adult muscle satellite cell niche. Cell.

[23] Smart N, Risebro CA, Melville AAD, Moses K, Schwartz RJ, Chien KR (2007). Thymosin β4 induces adult epicardial progenitor mobilization and neovascularization. Nature.

[24] Hastie ND (2017). Wilms’ tumour 1 (WT1) in development, homeostasis and disease. Development.

[25] Epelman S, Liu PP, Mann DL (2015). Role of innate and adaptive immune mechanisms in cardiac injury and repair. Nat Rev Immunol.

[26] Bollini S, Vieira JMN, Howard S, Dubè KN, Balmer GM, Smart N (2014). Re-activated adult epicardial progenitor cells are a heterogeneous population molecularly distinct from their embryonic counterparts. Stem Cells Dev.

[27] von Gise A, Zhou B, Honor LB, Ma Q, Petryk A, Pu WT (2011). WT1 regulates epicardial epithelial to mesenchymal transition through β-catenin and retinoic acid signaling pathways. Dev Biol.

[28] Messina E, Angelis LD, Frati G, Morrone S, Chimenti S, Fiordaliso F (2004). Isolation and expansion of adult cardiac stem cells from human and murine heart. Circ Res.

[29] Zhou B, Ma Q, Rajagopal S, Wu SM, Domian I, Rivera-Feliciano J (2008). Epicardial progenitors contribute to the cardiomyocyte lineage in the developing heart. Nature.

[30] Lo Furno D, Mannino G, Cardile V, Parenti R, Giuffrida R (2016). Potential therapeutic applications of adipose-derived mesenchymal stem cells. Stem Cells Dev.

[31] Gnecchi M, Zhang Z, Ni A, Dzau VJ (2008). Paracrine mechanisms in adult stem cell signaling and therapy. Circ Res.

[32] Gallo S, Sala V, Gatti S, Crepaldi T (2015). Cellular and molecular mechanisms of HGF/met in the cardiovascular system. Clin Sci.

[33] Wolf D, Li J, Ley K (2015). HGF guides T cells into the heart. Immunity.

[34] Arechederra M, Carmona R, González-Nuñez M, Gutiérrez-Uzquiza A, Bragado P, Cruz-González I (1832). Met signaling in cardiomyocytes is required for normal cardiac function in adult mice. Biochim Biophys Acta.

[35] Duan H-F, Wu C-T, Wu D-L, Lu Y, Liu H-J, Ha X-Q (2003). Treatment of myocardial ischemia with bone marrow-derived mesenchymal stem cells overexpressing hepatocyte growth factor. Mol Ther J Am Soc Gene Ther.

[36] Urbanek K, Rota M, Cascapera S, Bearzi C, Nascimbene A, Angelis AD (2005). Cardiac stem cells possess growth factor-receptor systems that after activation regenerate the infarcted myocardium, improving ventricular function and long-term survival. Circ Res.

[37] Jayasankar V, Woo YJ, Bish LT, Pirolli TJ, Chatterjee S, Berry MF (2003). Gene transfer of hepatocyte growth factor attenuates postinfarction heart failure. Circulation.

[38] Ono K, Matsumori A, Shioi T, Furukawa Y, Sasayama S (1997). Enhanced expression of hepatocyte growth factor/c-met by myocardial ischemia and reperfusion in a rat model. Circulation.

[39] Yamaguchi T, Sawa Y, Miyamoto Y, Takahashi T, Jau CC, Ahmet I (2005). Therapeutic angiogenesis induced by injecting hepatocyte growth factor in ischemic canine hearts. Surg Today.

[40] Martínez-Estrada OM, Lettice LA, Essafi A, Guadix JA, Slight J, Velecela V (2010). Wt1 is required for cardiovascular progenitor cell formation through transcriptional control of snail and E-cadherin. Nat Genet.

[41] Scholz H, Wagner K-D, Wagner N (2009). Role of the Wilms’ tumour transcription factor, Wt1, in blood vessel formation. Pflüg Arch - Eur J Physiol.

[42] Ocampo A, Reddy P, Martinez-Redondo P, Platero-Luengo A, Hatanaka F, Hishida T (2016). In vivo amelioration of age-associated hallmarks by partial reprogramming. Cell.

